# Microsatellite Instability-High, Malignant Insulinoma With Brain Metastasis

**DOI:** 10.7759/cureus.16969

**Published:** 2021-08-07

**Authors:** Jason Starr, Guillermo Puebla, Jessica McMillan, Jason T Lewis, Pashtoon M Kasi

**Affiliations:** 1 Hematology/Oncology, Mayo Clinic, Jacksonville, USA; 2 Hematology/Oncology, University of Puerto Rico, Medical Sciences Campus, San Juan, PRI; 3 Clinical Genomics, Mayo Clinic, Jacksonville, USA; 4 Department of Laboratory Medicine and Pathology, Mayo Clinic, Jacksonville, USA; 5 Medical Oncology, University of Iowa, Iowa City, USA

**Keywords:** pancreatic neuroendocrine tumors, pancreatic insulinoma, msi- high, cancer-immunotherapy, men 1

## Abstract

Insulinomas are the most common type of functional pancreatic neuroendocrine tumor. Although insulinomas usually are noninvasive or benign, 10% are deemed invasive or malignant. The pathologic mechanisms that lead to the malignant phenotype are not well elucidated. In this case report, we present a patient with stage 4 malignant insulinoma with metastasis to the liver, bone, and brain. Genetic analysis of the tumor showed that the tumor was mismatch-repair deficient and had a high rate of microsatellite instability. There was loss of *MLH1*- and *PMS2*-encoded protein expression, and *MLH1* and *MEN1* variants were identified. Notably, the liver metastasis showed considerable tumor heterogeneity (well differentiated) compared with the brain metastasis (poorly differentiated).

## Introduction

Insulinomas are pancreatic neuroendocrine tumors (panNETs) that originally were thought to originate from islet cells (β cells), however, more recent studies have suggested that the progenitor cell is from the acinar/ductal system [1]. Although most panNETs are nonfunctional, insulinomas account for approximately 60% of the functional panNETs [2,3]. Patients can present with the classic Whipple triad, which consists of symptoms of hypoglycemia, documented low blood sugar at the time of symptoms, and reversal of symptoms with glucose administration. Symptoms of hypoglycemia can include confusion, visual changes, and unusual behavior (e.g. staring off into space). Further, hypoglycemia results in the activation of the sympathetic-adrenal axis which can result in diaphoresis, palpitations, and tremulousness. At diagnosis, approximately 90% of insulinomas are localized and are considered benign [2,4]. PanNETs are classified on the basis of morphologic differentiation and proliferation rate (Ki-67 and mitotic index) [5].

Most panNETs are nonhereditary (sporadic) tumors, but approximately 10% are attributable to inherited disorders such as multiple endocrine neoplasia type 1 (MEN1 syndrome), von Hippel-Lindau syndrome, neurofibromatosis type 1, or tuberous sclerosis [6]. MEN1 syndrome is a hereditary condition characterized by development of multiple tumors in hormone-producing glands such as the parathyroid glands, anterior pituitary gland, and pancreas.

Patients with MEN1 syndrome have an 80% to 100% chance of panNET development, and 20% of these panNETs will be an insulinoma [4]. Because this disease is typically localized, the mainstay of treatment is surgical resection. The 10-year risk of recurrence is higher for patients with MEN1 syndrome (20% for patients with MEN1, 5% for patients without MEN1). For patients with malignant (ie, metastatic) insulinoma, the 10-year survival rate is approximately 30%, whereas it is approximately 90% for patients with localized disease [7].

## Case presentation

We describe a case of malignant insulinoma that is notable for the following unique aspects: the tumor was mismatch-repair deficient and microsatellite instability−high (dMMR/MSI-high), the patient had MLH1 (somatic) and MEN1 (germline) sequence variants, and a brain metastasis was identified with a gallium 68 (68Ga) DOTATATE positron emission tomography−computed tomography (PET-CT) scan. The reporting of this clinical case is in compliance with the CARE guidelines [8].

**Figure 1:**
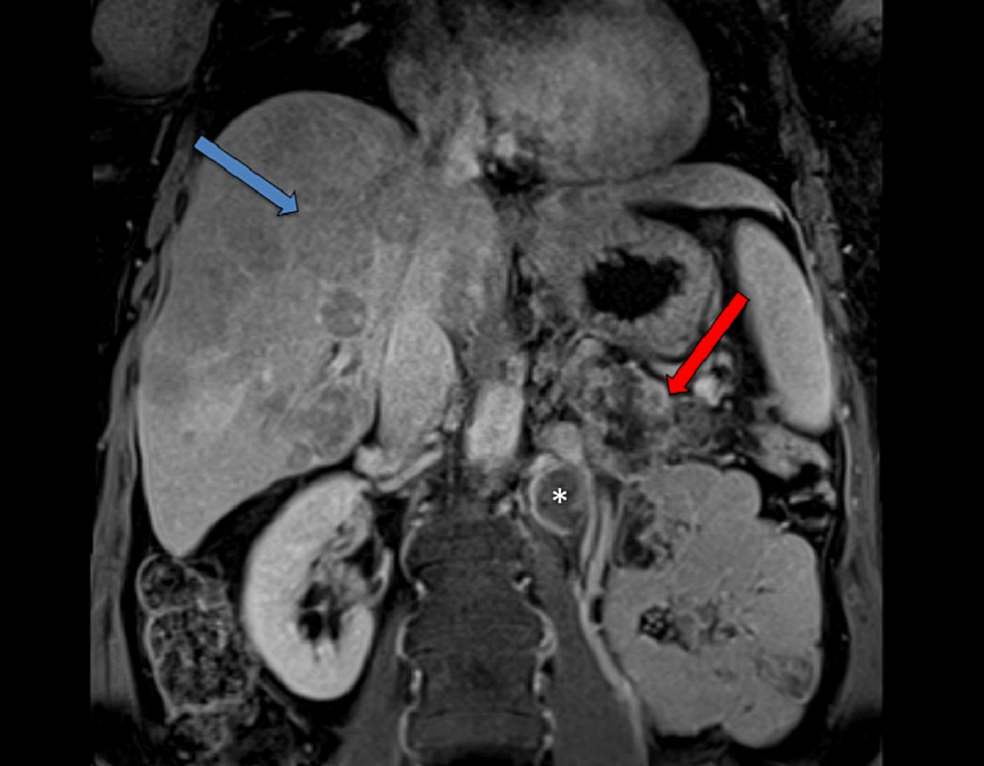
Magnetic resonance imaging (coronal) shows a pancreatic tail mass (red arrow), liver metastasis (blue arrow), and retroperitoneal lymphadenopathy (asterisk).

A 59-year-old White woman presented to the emergency department with the chief concern of refractory hypoglycemia. A physical examination showed unremarkable findings. Pertinent laboratory test results included a glucose level of 46mg/dL (reference range, 70-100); chromogranin A, 1,559ng/mL (reference, <93); insulin, 27.1mcIU/mL (reference, 2.6-24.9); and C-peptide, 6.5ng/mL (reference, 1.1-4.4). A magnetic resonance image of the abdomen with contrast showed a pancreatic tail mass, along with innumerable liver lesions and retroperitoneal lymphadenopathy (Figure 1).

**Figure 2:**
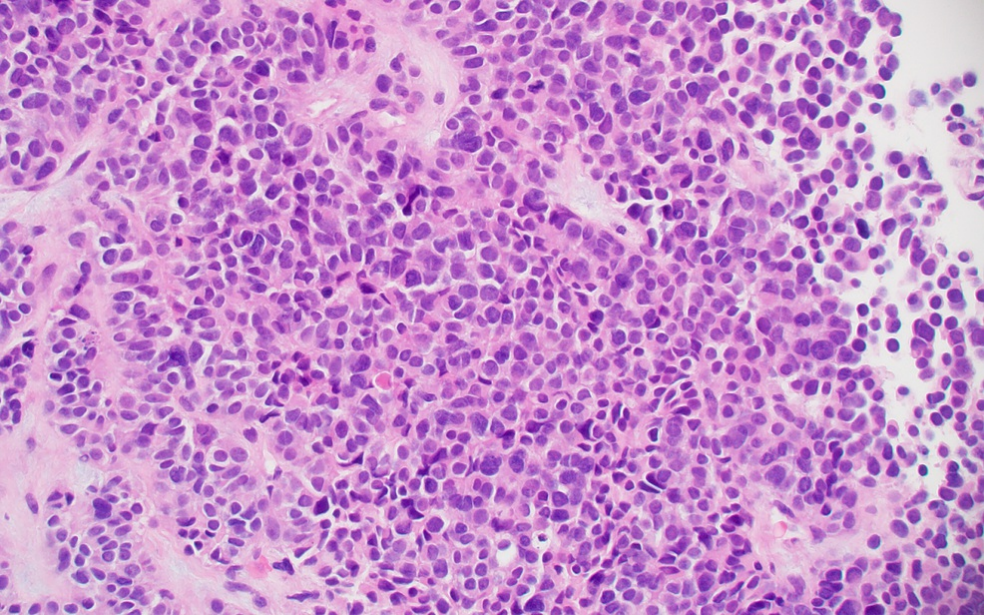
Liver, well-differentiated neuroendocrine tumor (hematoxylin-eosin, original magnification x 200).

**Figure 3:**
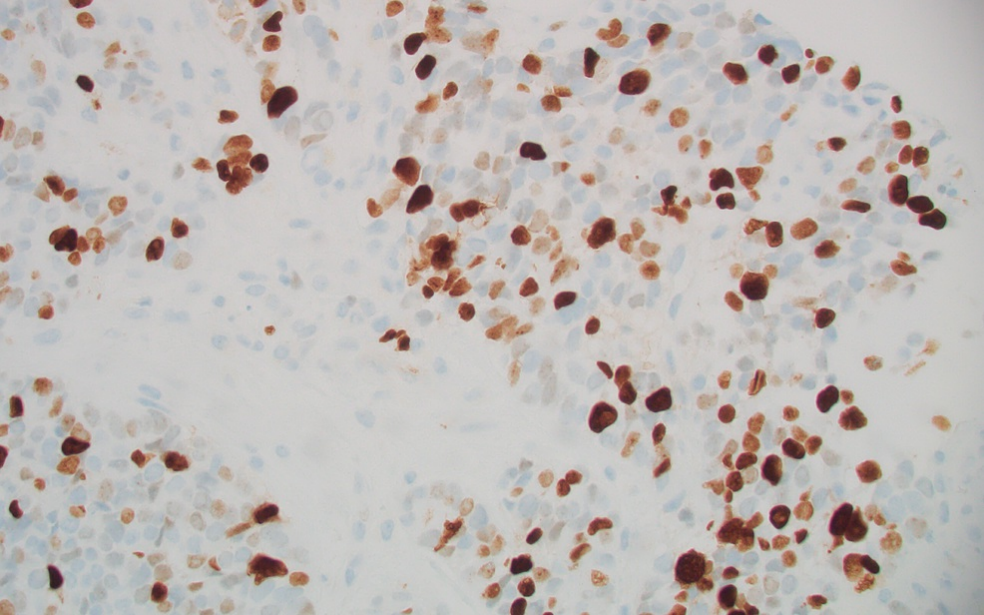
Liver, Ki-67 immunohistochemical stain (original magnification x 400). The proliferation rate was estimated to be 30%.

A biopsy specimen, taken from a liver lesion, showed a well-differentiated neuroendocrine tumor with a Ki-67 of 30% (grade 3) (Figures 2, 3).

Given the patient’s hypoglycemia, she received intravenous infusions of 10% glucose and octreotide, and diazoxide treatment was initiated. That same day, an interventional radiologist performed a bland embolization to the right lobe of the liver. The patient’s blood glucose level subsequently stabilized, and she was discharged.

**Figure 4:**
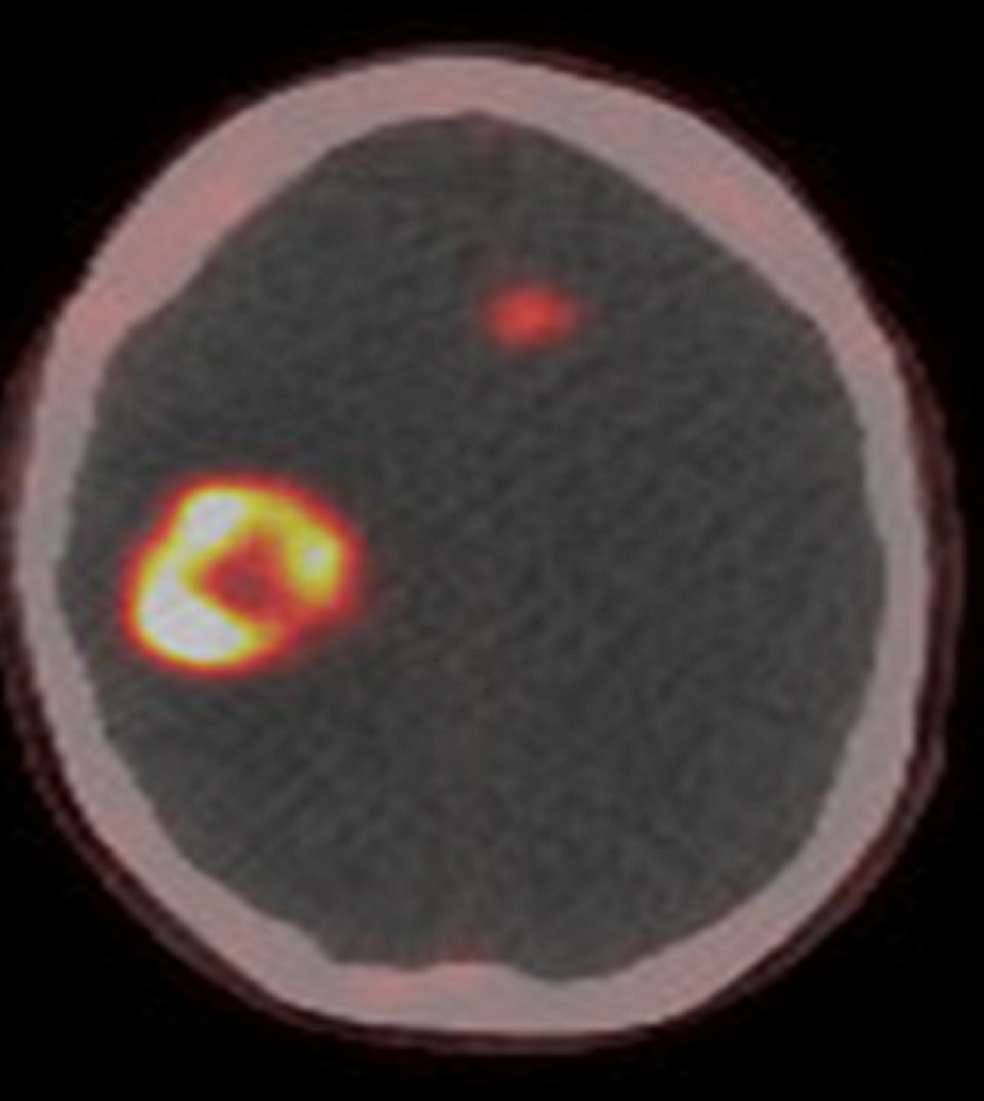
68Gallium DOTATATE positron emission tomography−computed tomography imaging (axial) of the brain shows a right parietal lesion and left frontal lesion.

**Figure 5:**
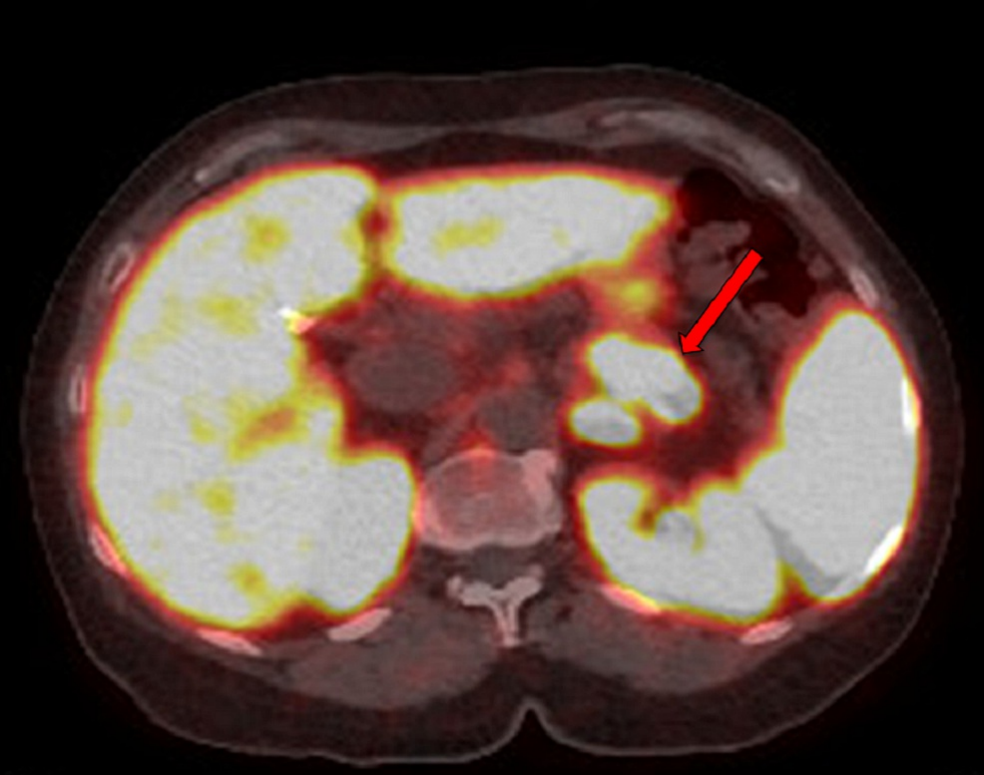
68Gallium DOTATATE positron emission tomography−computed tomography imaging (axial) shows a pancreatic tail lesion (red arrow).

In the clinic, the patient started treatment with 5-fluorouracil, leucovorin, and oxaliplatin (mFOLFOX6). The patient achieved a partial response, but progressive sensorimotor peripheral neuropathy developed after five months and mFOLFOX6 was discontinued. The patient had one cycle of capecitabine and temozolomide, but she had severe cytopenia and rapid progression of disease in the abdomen. A 68Ga DOTATATE PET-CT scan was obtained and identified a large right parietal brain tumor (approximately 3.3 cm) with associated edema, mass effect, and mild midline shift, along with a left frontal lobe lesion (Figure 4). The scan also showed a pancreatic tail lesion, hepatic metastasis, and uptake in the right pelvis (Figure 5).

**Figure 6:**
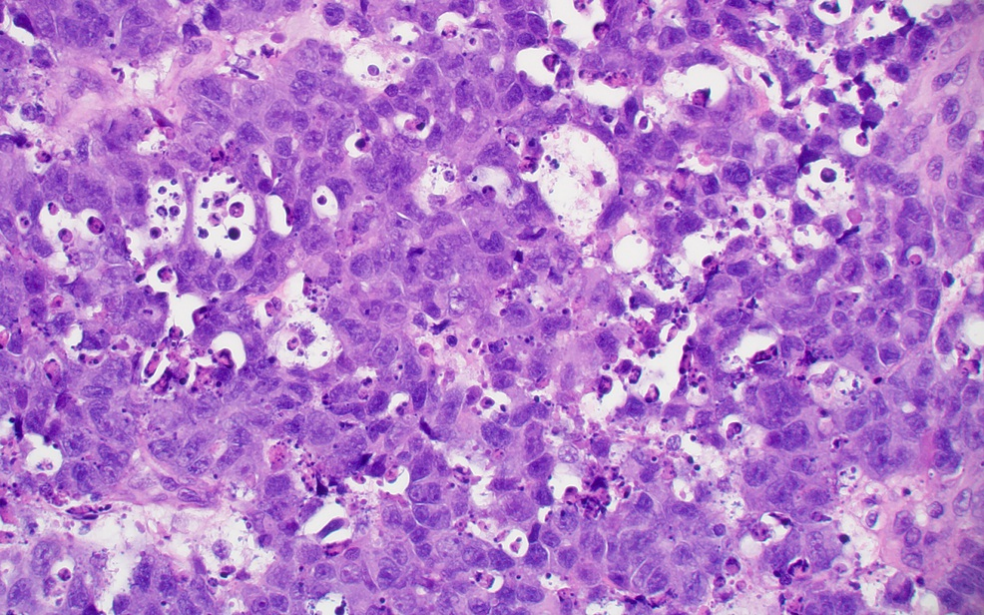
Brain, poorly differentiated neuroendocrine tumor (hematoxylin-eosin, original magnification x 400).

**Figure 7:**
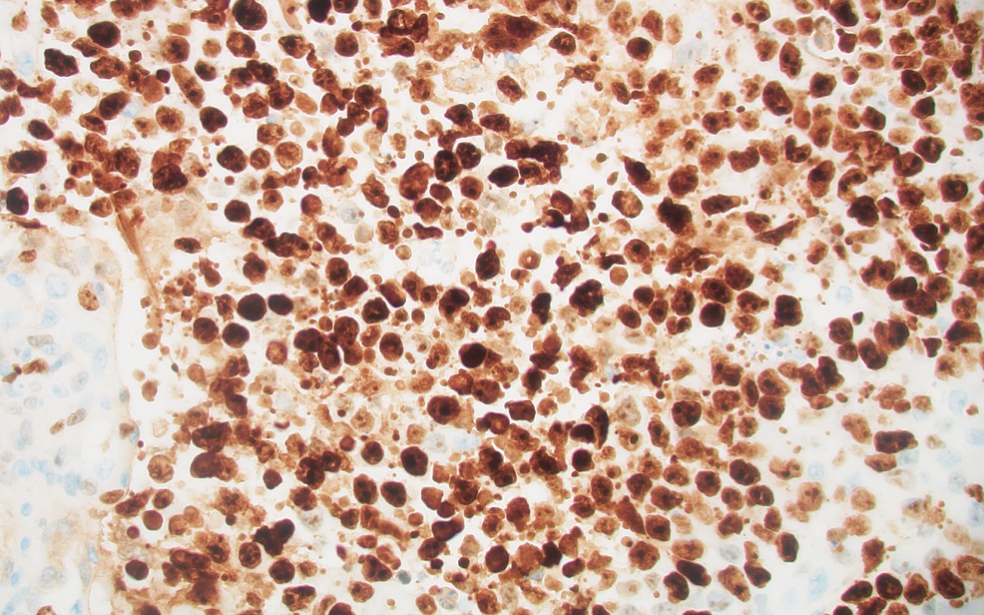
Brain, Ki-67 immunohistochemical stain (original magnification x 400). The proliferation rate was estimated to be 90%.

She underwent a right craniotomy and resection of the metastatic lesion. Pathologic analysis showed a poorly differentiated neuroendocrine carcinoma with a Ki-67 of 90% (Figures 6, 7).

Next-generation sequencing of the original liver biopsy specimen was performed after the surgery and showed pathologic sequence variations in the MEN1 and MLH1 genes (Table 1).

**Table 1:** Genetic Features of the Malignant Insulinoma

Pathogenic sequence variant	Protein alteration	Exon	DNA alteration
MEN1 (germline)	R516fs	10	c.1546delC
MLH1 (somatic)	K255fs	9	c.762_763delGA

**Figure 8:**
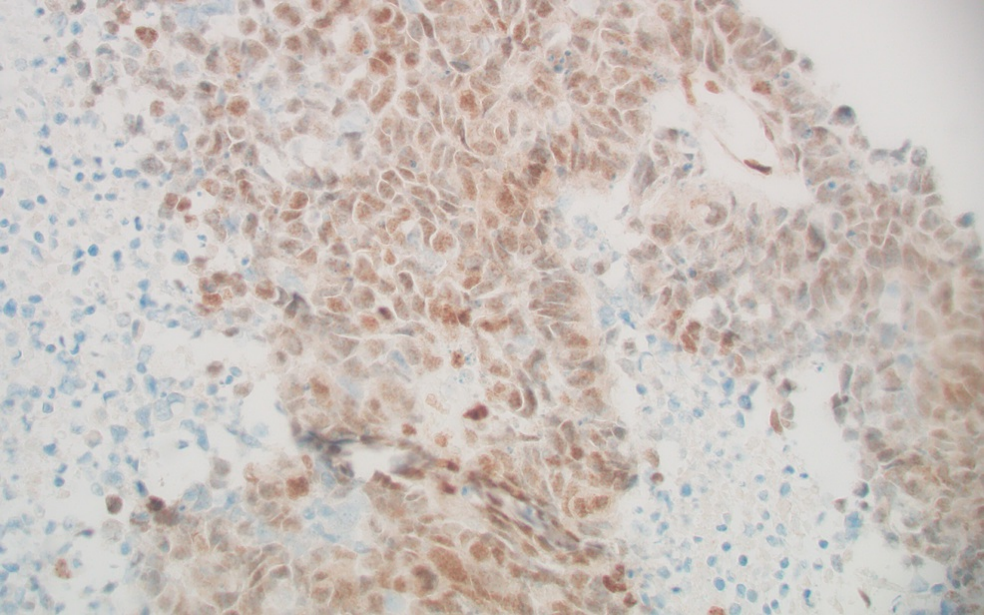
MLH1, weakly positive (+1).

**Figure 9:**
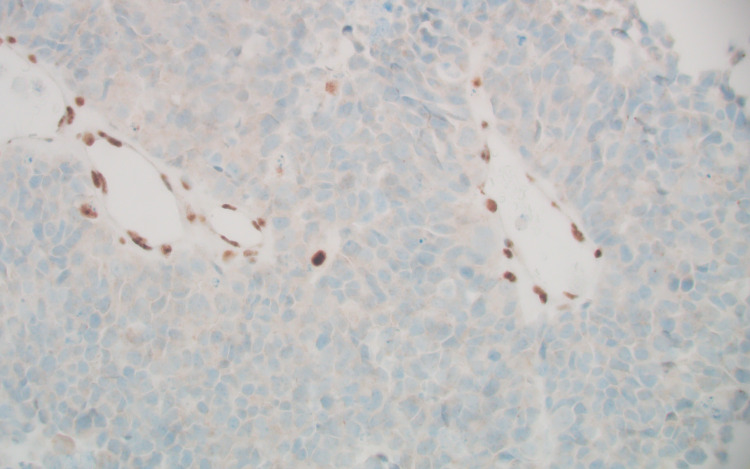
PMS2, absent.

**Figure 10:**
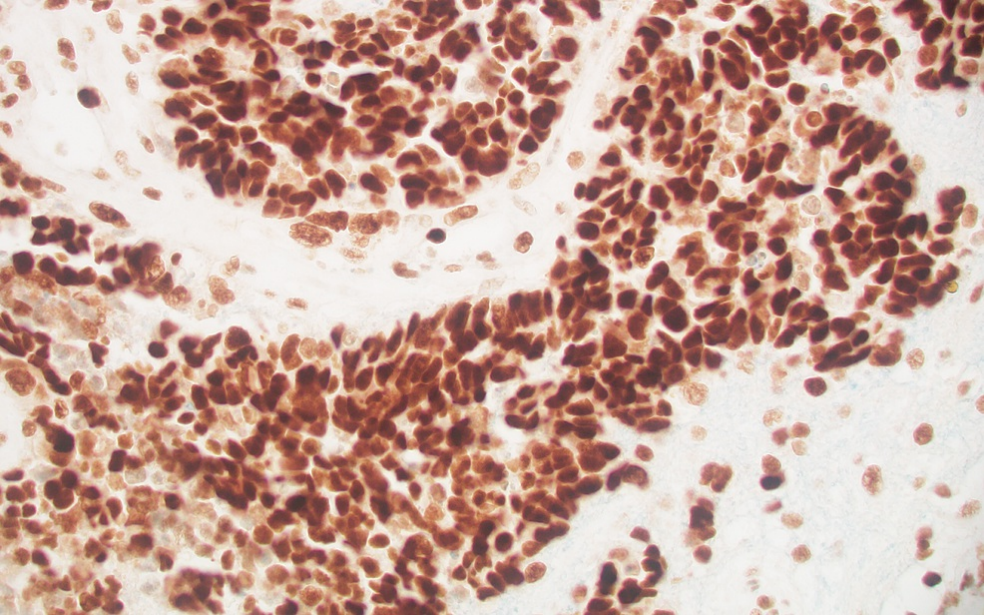
MSH2, positive (+2).

**Figure 11:**
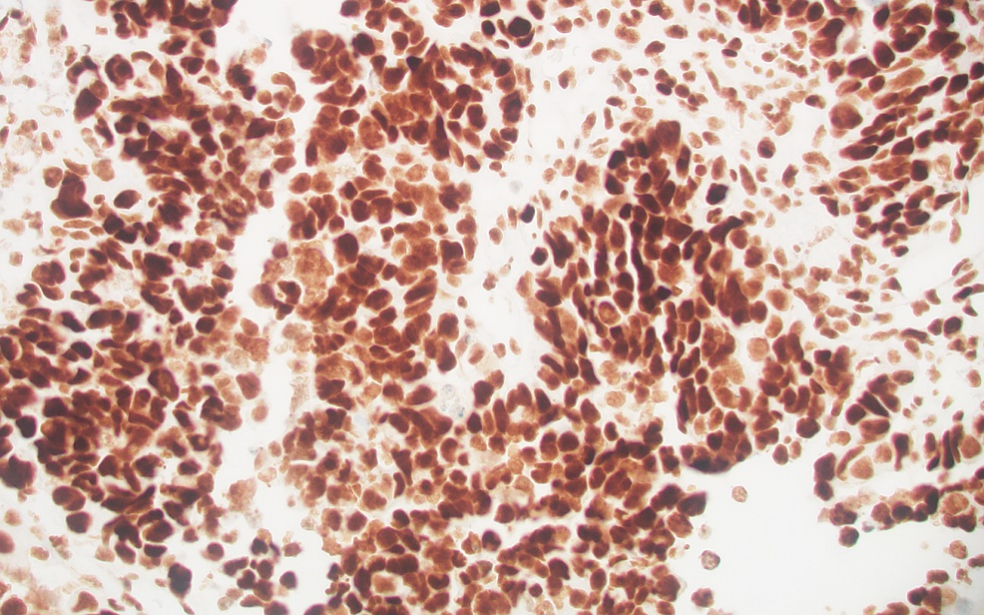
MSH6, positive (+2).

The tumor had an intermediate mutational burden (eight variants/Mb). Immunohistochemical (IHC) analysis of the tumor showed loss of MLH1 protein expression (weakly positive, +1) but no PMS2 expression. MSH6 and MSH2 protein expression were normal at 2+ (Figures 8, 9, 10, 11).

Immunostaining with the SP142 antibody showed no PD-L1 expression. Additionally, polymerase chain reaction testing confirmed the MSI-H status. Of note, the brain pathology specimen was not sent for next-generation sequencing.

Given the molecular findings of dMMR/MSI-H, the patient promptly started treatment with the anti-PD-1 antibody, pembrolizumab. The patient received two doses, but unfortunately, her clinical status quickly declined because of increasing abdominal pain, progressive weakness, and failure to thrive. The patient entered hospice care and died shortly thereafter.

## Discussion

Genetics of MEN1, MLH1, and MSI-H

Using multiplex, next-generation sequencing, we identified pathologic frameshift sequence variations in MEN1 (germline variant) and MLH1 (somatic variant) (Table 1). The MEN1 gene, first discovered in 1988, is located on chromosome 11q13 and encodes the protein menin. MEN1 is a tumor-suppressor gene involved in cell cycle regulation, but the exact mechanism of tumorigenesis in MEN1 variants is not completely understood. Somatic variants of MEN1 have been identified in several nonhereditary tumors such as parathyroid adenomas, gastrinomas, and insulinomas [9,10]. Loss of heterozygosity of MEN1 can lead to MEN1 syndrome. Notably, 25% to 44% of MEN1 variants identified in panNETs are somatic in nature [11-13]. The variant identified in our patient is a known germline disease-associated allele (ClinVar database: https://www.ncbi.nlm.nih.gov/clinvar/variation/200999/). Our patient did not have a known family history of MEN1 syndrome.

MLH1 is part of a gene family (which includes MSH2, MSH3, MSH6, and PMS2) that is responsible for the surveillance and repair of DNA base mismatches (ie, mismatch repair), in which loss leads to dysregulated DNA repair. The human genome has long stretches of conserved repetitive DNA sequences that help protect the coding regions. These stretches are prone to variation, and thus, if mismatch repair is dysregulated, errors can accumulate and ultimately result in MSI-H. This faulty DNA repair is best exemplified by Lynch syndrome, an inherited autosomal dominant disease, in which variants in mismatch repair genes lead to increased risk of various malignancies, most commonly colon and uterine cancer. Studies have shown that up to 36% of panNET cases have MLH1 loss. One mechanism by which this occurs is hypermethylation of the gene [14,15]. Mei et al. studied 55 sporadic insulinomas and showed that 20 (36%) showed reduced expression of MLH1 (defined as <25% positive with IHC staining) or loss of MLH1 (determined with IHC), which corresponded to a 33% rate of MSI-H [16].

Detection of brain metastasis with 68Ga DOTATATE PET-CT

Brain metastasis of well-differentiated neuroendocrine tumors is rare, affecting an estimated 1.5% to 5% of cases [16]. In our review of the literature, we identified only two prior reports of insulinoma with brain metastasis [17,18]. The 68Ga DOTATATE PET-CT scan has dramatically increased the ability to detect previously occult disease [19]. The sensitivity of this imaging technique is shown in our case because it identified intracranial disease (brain metastasis) in a patient who had no neurologic symptoms at the time. It should be noted that meningiomas also have somatostatin expression and thus will exhibit uptake, which could lead to a false-positive result in a patient with neuroendocrine disease.

## Conclusions

This case report describes a patient with a malignant insulinoma with some unique features, including MEN1 and MLH1 sequence variants, dMMR/MSI-H status from loss of MLH1, and identification of brain metastasis with 68Ga DOTATATE PET-CT. It is important to point out the heterogeneity of the tumor, which presented as a well-differentiated neuroendocrine tumor and later manifested with poorly differentiated morphology in the brain. Also of interest was the lack of response to pembrolizumab, even though the tumor was dMMR/MSI-H. Based on the above case and the available literature, we recommend testing patients with panNETs, specifically insulinomas, for the presence or absence of MMR proteins or MSI status.
